# The dangers of hemilithotomy positioning on traction tables: case report of a well-leg drop foot after contralateral femoral nailing

**DOI:** 10.1186/s13037-015-0069-2

**Published:** 2015-05-14

**Authors:** Kai-Lan Hsu, Chih-Wei Chang, Chii-Jeng Lin, Chih-Han Chang, Wei-Ren Su, Shu-Min Chen

**Affiliations:** Division of Orthopaedics, Department of Surgery, National Cheng Kung University Hospital, Dou-Liou Branch, National Cheng Kung University, Douliou, Taiwan; Department of Orthopedics, National Cheng Kung University Hospital, No 138, Sheng-Li Road, Tainan City, 70428 Taiwan; Division of Traumatology, National Cheng Kung University Medical Center, Tainan, Taiwan; Department of Biomedical Engineering, National Cheng Kung University, Tainan, Taiwan; Department of Physical Medicine & Rehabilitation, National Cheng Kung University Medical Center, Tainan, Taiwan

**Keywords:** Drop foot, Contralateral leg, Morbidity, Hemilithotomy position, Peroneal palsy

## Abstract

**Background:**

Postoperative contralateral morbidities after fracture fixation surgery by hemilithotomy positioning on traction table is uncommon. We’d report a case of unexpected common peroneal nerve palsy developed on the contralateral side manifesting with drop foot after a common orthopedic femoral nailing.

**Case report:**

A 28-year-old female sustained an unusual common peroneal nerve palsy manifesting contralateral drop foot after prolonged femoral nailing. Although the initial presentations were similar to the notorious well-leg compartment syndrome, a benign course with complete recovery in functions was observed 3 months later. After neurophysiologic exam and review of pertinent literature, this iatrogenic and transient dysfunction was delineated to be position-related neuropraxia.

**Conclusion:**

Position adjustment at intervals or complete avoidance of prolonged knee hyperflexion is recommended to prevent contralateral common peroneal nerve morbidity.

## Background

Ipsilateral morbidities after fracture fixation are often related to either initial trauma to the involved neurovascular structures or secondary events caused by interventional procedures or devices. By contrast, postoperative contralateral morbidities are far less encountered; hence, little or no attention is paid during surgery. However, during a lengthy surgical procedure, unexpected complications related to patient positioning may insidiously develop even on a healthy extremity [1]. Thus, surgeons have to be aware of this potential morbidity while carrying out surgical procedures.

This report documents an unexpected common peroneal nerve palsy developed on the contralateral side manifesting with drop foot after a common orthopedic femoral nailing. Fortunately, this functional impairment was transient and completely recovered after a 3-month conservative treatment. A careful review of this case and pertinent literature indicated that the risk from faulty positioning to consequent common peroneal nerve palsy should be highlighted.

## Case presentation

**Fig. 1:**
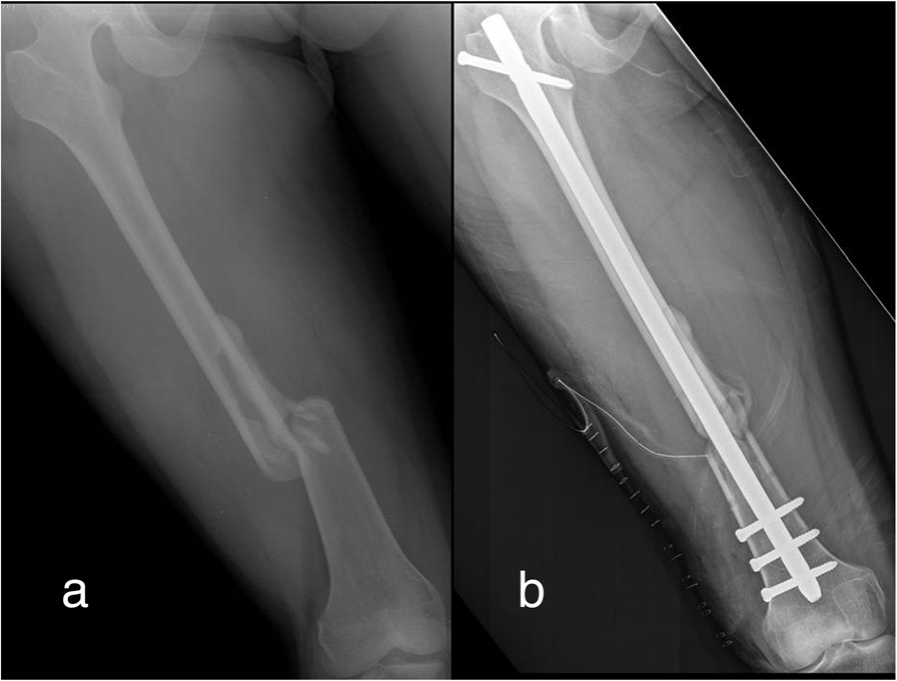
(**a**) Radiograph of the femur showing a comminuted, spiral fracture over the shaft before operation; and (**b**) a satisfactory fixation was obtained after a lengthy closed femoral nailing

**Fig. 2:**
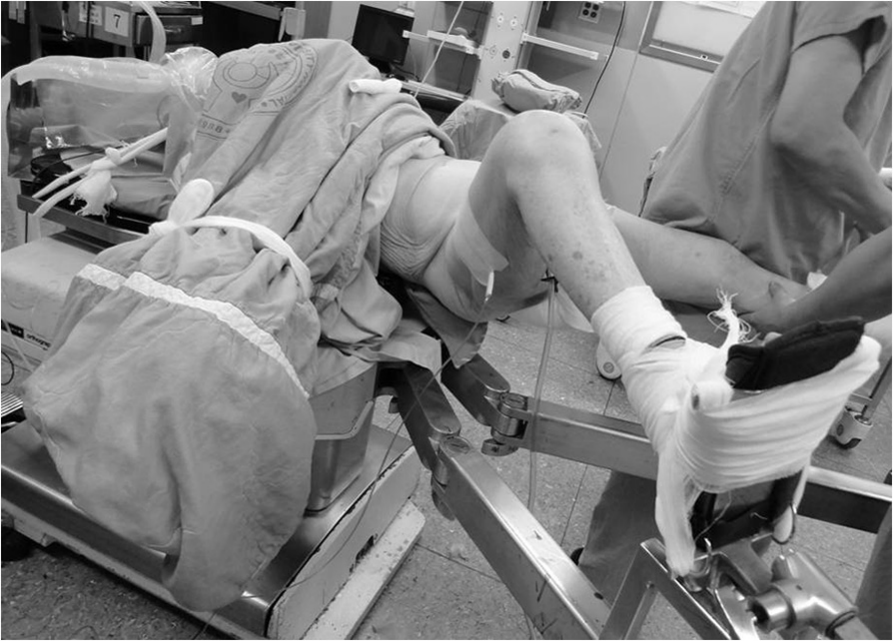
A supine hemilithotomy position in authors’ institute is routinely maintained with a boot immobilization and patients’ knee flexed over 90° instead of a calf supporter or a suspension knee strap, making the posture of the non-operated limb similar to squatting

A 28-year-old female (body mass index of 20.2 kg/m^2^) sustained a comminuted, spiral fracture over her right femur in a motor vehicle accident (Fig. 1a). On physical examination in the emergency room, she was alert and oriented, with a healthy contralateral leg. Initial neurovascular examinations of both legs were normal. The fracture was then treated with closed reduction and stabilization using an 11 × 380 mm intramedullary nail (Targon®; Aesculap, Tuttlingen, Germany). The patient underwent these surgical procedures under general anesthesia, and was kept in a supine hemilithotomy position- the non-operated leg was held by a boot and positioned in 80° of hip flexion, 30° of abduction, and 105° of knee flexion without any leg holders or fixation straps around the knee (Fig. 2). Because of technical difficulties in aligning fragments closely as well as in locking distal static screws, it took four hours to obtain a satisfactory fixation (Fig. 1b).

When the patient regained consciousness, she started to complain a “dead” foot with difficulties in movement and severe numbness in the non-operated leg. There was no swelling, local heat and cyanosis over her left leg. At 24 h after surgery, however, she already had a left drop foot manifesting complete loss of ankle dorsi-flexion as well as impaired sensation below the knee, especially at the dorsum of the foot. In the remaining stay, the motor strength of the involved leg did not improve, although her sensory examination did evolve to a classic common peroneal palsy distribution. With symptoms compatible with a common peroneal nerve palsy, we fitted the patient with an ankle-foot orthosis and administered physiotherapy. Three weeks later, scheduled neurophysiologic studies were performed. The motor nerve conduction velocity (NCV) revealed decreased amplitude in left common peroneal nerve and needle electromyography (EMG) showed no motor unit action potential (MUAP) in left tibialis anterior muscle, which was compatible with left common peroneal neuropathy.

After discharge, she had regular outpatient follow-ups at our rehabilitation department, and was taught home exercises and given electrical stimulation. Both the motor and sensory functions of her left leg showed gradual improvement. At two months after the event she was nearly ready to discard her orthoses, and a complete recovery of the non-operated leg without permanent sequelae was observed at the 3-month follow-up.

## Discussion

Because of the inherent anatomical or physiological susceptibility, the common peroneal nerve is frequently involved in lower extremities traumas, and commonly presents as foot drop once injured because of the paresis of its distributing ankle dorsiflexor, tibialis anterior, toe dorsiflexors, extensor digitorum brevis and extensor hallucis longus muscles. In the literature, postoperative drop foot on the contralateral, uninjured leg is rare and only exists in case reports. Tait and Danton [2] reported two sciatic nerve palsies in the contra-lateral leg after femoral nailing, which were speculated by position-related excessive stretching.

Several causes contribute to common peroneal nerve neuropathy, including external compression (Plaster cast, brace or immobilization), direct trauma, traction injury, and entrapment in the fibular tunnel [3,4]. In unilateral lithotomy position, direct external compression by calf support may cause common peroneal neuropathy and more serious clinical presentation known as the “well-leg acute compartment syndrome” [1,5–9]. The flexed knee and calf support significantly increase intramuscular pressures (direct compression theory) and decreases ankle blood pressure (vascular insufficiency theory) [10]. The combination of both pathophysiologic events results in local ischemia, tissue edema, than acute compartment syndrome and neuropathy may happen. In our case, a boot support rather than calf support was used to prevent direct compression around the knee. Besides, there were no other indicative symptoms of compartment syndrome such as local swelling and ischemia pain complained. Thus, the contributing mechanism to our case should not be identical to that of the “well-leg acute compartment syndrome”.

Prolonged unhealthy position is another cause of common peroneal neuropathy, which induces a nerve entrapment syndrome presenting as a foot drop. In Asian culture, certain positions with knee flexion like squatting, kneeling or habitual leg crossing while seated are frequently required. Several reports mentioned related neuropathy developed after prolonged maintenance of these positions [11,3,12]. In 2004, Sangwan et al. [13] reported 34 peroneal neuropathies in 30 farmers after a prolonged squatting (>5 h). All the patients made a complete recovery without surgical interventions. In 2013, Yu et al. also reported 26 cases of posture induced common peroneal nerve, and 14 of them occurred after long time squatting (average 124.2 min). During squatting, the peroneal nerve is compressed between biceps tendon, lateral head of gastronemius muscle and fibular head [3]. Thus, prolonged squatting may cause postural peroneal nerve palsy.

The hemilithotomy position in our patients is routinely maintained with a boot immobilization, with the patients’ knee flexed over 90° without any suspension by a calf supporter or a knee strap, making the posture similar to squatting. Moreover, we also noted that the shared features reported in the literature, including of a similar prolonged unhealthy posture, slender body shape, and even the benign courses, were compatible with our case.

In acute onset neuropathies, detailed clinical examination and NCV/EMG study in the relative early period (3 weeks after the injury) are advocated to assess the severity and capacity for possible spontaneous recovery [14–16]. In peroneal nerve palsies induced by posture, the most common nerve injury is neuropraxia, and a benign course with good recovery within several weeks would be achieved. In our case, gradual improvement in sensory functions without active demyelination changes in subsequent neurophysiologic studies indicated a transient dysfunction with neuropraxia, the most benign and reversible nerve injury.

## Conclusion

In conclusion, we report an unusual case of contralateral common peroneal nerve palsy, which manifested drop foot, developed after femoral nailing. A possible iatrogenic neuropathy secondary to position-related compression is speculated as the cause. Our case highlights the need to monitor patient positioning even during the most common orthopedic procedures. Although satisfactory recovery may be obtained, as our case, unexpected dysfunction of the uninjured leg and even resulted litigations warrant surgeons’ awareness of the potential morbidity to the uninjured leg during lengthy procedures. We recommend considerable adjustment, allowing the release of patients’ extremities at regular intervals under prolonged hemilithotomy position (similar to the pneumatic tourniquet), or alternative surgical approaches in predictably lengthy operations to avoid potential complications.

## Consent

Written informed consent was obtained from the patient for publication of this Case report and any accompanying images. A copy of the written consent is available for review by the Editor-in-Chief of this journal.

## Abbreviation

EMG: Electromyography
NCV: Nerve conduction velocity

## References

[1] Flierl MA, Stahel PF, Hak DJ, Morgan SJ, Smith WR (2010). Traction table-related complications in orthopaedic surgery. J Am Acad Orthop Surg.

[2] Tait GR, Danton M (1991). Contralateral sciatic nerve palsy following femoral nailing. J Bone Joint Surg Br.

[3] Stewart JD (2008). Foot drop: where, why and what to do?. Pract Neurol.

[4] Masakado Y, Kawakami M, Suzuki K, Abe L, Ota T, Kimura A (2008). Clinical neurophysiology in the diagnosis of peroneal nerve palsy. Keio J Med.

[5] Mathews PV, Perry JJ, Murray PC (2001). Compartment syndrome of the well leg as a result of the hemilithotomy position: a report of two cases and review of literature. J Orthop Trauma.

[6] Meena S, Trikha V, Saini P, Kumar N, Kr S (2014). Well-leg compartment syndrome after fracture fixation in hemilithotomy position: case report of a preventable condition. Med Princ Pract.

[7] Noordin S, Allana S, Wajid (2009). Well leg compartment syndrome: the debit side of hemilithotomy position. J Ayub Med Coll Abbottabad.

[8] Tan V, Pepe MD, Glaser DL, Seldes RM, Heppenstall RB, Esterhai JL (2000). Well-leg compartment pressures during hemilithotomy position for fracture fixation. J Orthop Trauma.

[9] Dugdale TW, Schutzer SF, Deafenbaugh MK, Bartosh RA (1989). Compartment syndrome complicating use of the hemi-lithotomy position during femoral nailing. A report of two cases. J Bone Joint Surg Am.

[10] Meyer RS, White KK, Smith JM, Groppo ER, Mubarak SJ, Hargens AR (2002). Intramuscular and blood pressures in legs positioned in the hemilithotomy position: clarification of risk factors for well-leg acute compartment syndrome. J Bone Joint Surg Am.

[11] Yu JK, Yang JS, Kang SH, Cho YJ (2013). Clinical characteristics of peroneal nerve palsy by posture. J Korean Neurosurg Soc.

[12] Togrol E (2000). Bilateral peroneal nerve palsy induced by prolonged squatting. Mil Med.

[13] Sangwan SS, Marya KM, Kundu ZS, Yadav V, Devgan A, Siwach RC (2004). Compressive peroneal neuropathy during harvesting season in Indian farmers. Trop Doct.

[14] Aprile I, Padua L, Padua R, D’Amico P, Meloni A, Caliandro P (2000). Peroneal mononeuropathy: predisposing factors, and clinical and neurophysiological relationships. Neurol Sci.

[15] Thoma A, Fawcett S, Ginty M, Veltri K (2001). Decompression of the common peroneal nerve: experience with 20 consecutive cases. Plast Reconstr Surg.

[16] Rempel DM, Diao E (2004). Entrapment neuropathies: pathophysiology and pathogenesis. J Electromyogr Kinesiol.

